# Mechanism Understanding for Size Regulation of Silver Nanowires Mediated by Halogen Ions

**DOI:** 10.3390/nano12152681

**Published:** 2022-08-04

**Authors:** Ni Xiao, Yinan Chen, Wei Weng, Xiaopeng Chi, Hang Chen, Ding Tang, Shuiping Zhong

**Affiliations:** 1 School of Materials Science and Engineering, Fuzhou University, Fuzhou 350108, China; 2 Zijin School of Geology and Mining, Fuzhou University, Fuzhou 350108, China; 3 Fujian Key Laboratory of Green Extraction and High-Value Utilization of Energy Metals, Fuzhou University, Fuzhou 350108, China; 4 Zijin Mining Group Co., Ltd., Shanghang 364200, China; 5 State Key Laboratory of Comprehensive Utilization of Low Grade Refractory Gold Ores, Shanghang 364200, China

**Keywords:** silver nanowires, halogen ions, aspect ratio, surface adsorption, density functional theory

## Abstract

The controllable preparation of silver nanowires (AgNWs) with a high aspect ratio is key for enabling their applications on a large scale. Herein, the aspect ratio regulation of AgNWs mediated by halogen ion composition in ethylene glycol system was systematically investigated and the size evolution mechanism is elaborately understood. The co-addition of Br^−^ and Cl^−^ results in AgNWs with the highest aspect ratio of 1031. The surface physicochemical analysis of AgNWs and the density functional theory calculations indicate that the co-addition of Br^−^ and Cl^−^ contributes to the much-enhanced preferential growth of the Ag(111) crystal plane. At the same time, when Cl^−^ and Br^−^ coexist in the solution, the growth of the Ag(100) crystal plane on the AgNWs was restrained compared with that in the single Cl^−^ system. Resultantly, the enhanced growth of Ag(111) and the inhibited growth of Ag(100) contribute to the formation of AgNWs with a higher aspect ratio in the Cl–Br mixed solution. The results can provide new insights for understanding the morphology and size evolution during the AgNWs preparation in ethylene glycol system.

## 1. Introduction

Silver nanowires (AgNWs) possesses excellent electrical, thermal, optical, and mechanical properties as well as extraordinary thermoelectric merit [1,2], thus being widely used in surface-enhanced Raman scattering (SERS) [3,4], transparent conductive film (TCF) [5], chemical/biological sensing [6,7], electromagnetic interference (EMI) shielding materials [8,9], flexible and stretchable electronic devices [10], etc. Specifically, the one-dimensional characteristics of AgNWs can facilitate the charge transfer and decrease the electrical resistance as well as promise high flexibility. The aspect ratio of AgNWs (ratio of length to diameter) is a critical parameter in providing highly conductive networks with good transparency for AgNWs-based films [11]. Additionally, AgNWs with a thinner diameter and higher aspect ratio are beneficial for the performance of the TCF, especially for high-end applications, such as touch panel screens that require low sheet resistance and low haze [12]. For example, Khanarian et al. [13] found that superfine Ag NWs (diameter < 50 nm, length > 5 μm) were required to achieve a high transmittance of >90% with a low sheet resistance ~100 Ω sq^−1^. Mutiso et al. [14] proposed that an aspect ratio as large as 800 was required for the high performance of AgNW TCFs with sheet resistance ~10 Ω sq^−1^ for a transparency of ~90% at 550 nm. Thinner AgNWs generate more “open area” for light to pass through, and higher aspect ratio decreased the number of high resistance wire contacts in the film [11,15]. Therefore, size regulation for preparing AgNWs with high aspect ratio is urgent needed [2,16].

The polyol method is one of the most commonly used processes for AgNWs preparation among the widely explored strategies, including template-assisted synthesis [17,18,19], electrochemical technique [20,21], the UV irradiation photo-reduction technique [22,23], hydrothermal synthesis [24,25,26], wet chemical synthesis [27], and polyol synthesis [28,29,30,31,32,33,34,35]. Polyol synthesis is highly desirable due to the following merits: (1) simple operation with high yield. AgNWs can be obtained by a facile reduction of AgNO_3_ in the ethylene glycol (EG) solution at about 160 °C, with the yield being high up to 97% [36]. (2) The easy regulation for AgNWs growth. The length and diameter of AgNWs can be easily tuned by changing the molecular weight of polyvinyl pyrrolidone (PVP), controlling the concentration of added agents or adjusting the agent species such as Fe^3+^, Cu^2+^, Cl^−^, and Br^−^ [29,30,33,34,35,36]. Nevertheless, further increasing the aspect ratio of AgNWs by the polyol process is an appealing but challenging task.

The formation of AgNWs with high aspect ratio relies on the preferential growth of the Ag(111) crystal plane than the Ag(100) crystal plane [34,35,37]. The preferential growth of the Ag(111) crystal plane can be realized by adding the PVP agent because the selectively adsorbed PVP on the Ag(100) crystal plane can retard the growth of Ag(100) plane [34,35,37,38]. However, the adhered PVP molecules on the AgNWs act as an insulating layer, weakening the application ability of AgNWs [31,39]. A redeeming strategy to regulate the aspect ratio of AgNWs is the addition of halogen ions, namely Cl^−^ and Br^−^, which can also tune the relative growth rate of Ag(111) and Ag(100) [2,36]. Compared with the PVP agent, the halogen ions can be easily removed by rinsing with ethanol, therefore posing negligible negative effects on the properties of AgNWs [2]. Deeply understanding the influence mechanism of halogen ions on AgNWs growth is of prime importance for elaborately tuning the morphology of AgNWs, yet still lacks consensus [36,40,41,42].

**Figure 1:**
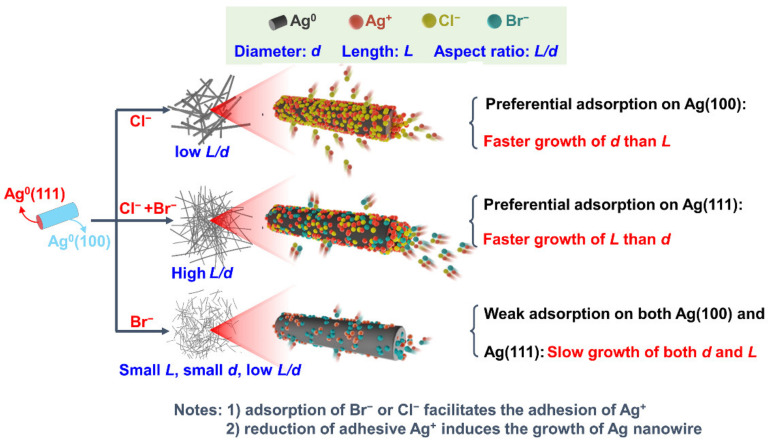
Proposed mechanism for the size regulation of AgNWs by halogen ions.

Herein, the synergy mechanism of Br^−^ and Cl^−^ on the formation of AgNWs with a high aspect ratio was investigated with the aid of surface chemistry analysis and adsorption energy calculation by density functional theory (DFT). As shown in Figure 1, the length increase of the AgNWs relies on the growth of Ag(111) crystal plane while diameter increase originates from the growth of Ag(100) plane. For single Cl^−^ addition, the preferential adsorption of Cl^−^ on Ag(100) crystal plane occurs. Resultantly, more Ag^+^ is accumulated on the Ag(100) plane due to the very low solubility of silver halides, which results in the faster growth of the diameter (*d*) than the length (*L*), leading to a relatively low aspect ratio (*L*/*d*). For single Br^−^ addition, adsorption of Br^−^ on both Ag(111) and Ag(100) is weak, which means that only a small amount of Ag^+^ are accumulated on both planes, resulting in very short and thin AgNWs products. For the co-addition of Br^−^ and Cl^−^, the halides are more strongly adsorbed on the Ag(111) crystal plane than that of Ag(100), which facilitates the faster growth of length than of diameter, contributing to the AgNWs with the highest aspect ratio. The results can provide new insights for understanding the AgNWs growth mechanism in the halogen-containing systems of the polyol method.

## 2. Materials and Methods

### 2.1. Materials

Silver nitrate (AgNO_3_, ≥99.8%), acetone (≥99.5%), NaCl (99.5%), NaI (99.5%), and absolute ethyl alcohol (≥99.5%) were purchased from Sinopharm Chemical Reagent Co., Ltd. (Shanghai, China). Polyvinylpyrrolidone (PVP) (*M*_w_ ≈ 40,000 g/mol) was purchased from Sigma-Aldrich (St. Louis, MO, USA). Ferric chloride hexahydrate (FeCl_3_·6H_2_O, ≥99.5%) was obtained from Kemiou Chemical Reagent Co., Ltd. (Tianjin, China). Ferric tribromide (FeBr_3_, 98%) was obtained from Macklin Biochemical Co., Ltd. (Shanghai, China). Ethylene glycol (EG, ≥99.5%) was acquired from Xilong Scientific Co., Ltd. (Shantou, China). Ultra-pure water obtained by a Direct-Q 3 UV water purification system (Millipore, Burlington, MA, USA) and used throughout. All chemicals were used as received without further purification.

### 2.2. Synthesis of Silver Nanowires

In a typical synthesis procedure, 40 mL EG and 2.489 g PVP (*M*_w_ ≈ 40,000 g/mol) were added into a 100 mL three-necked flask placed in an oil bath at room temperature. The flask was slowly heated to 160 °C within 40 min with vigorous magnetic stirring. The flask was sealed with frosted glass caps. When the temperature was stable at 160 °C for 1 h, 200 μL of 0.05 M control agent, i.e., FeCl_3_, FeBr_3_, or their mixture in EG, was added into the flask. After ten minutes, 1 mL EG solution containing 0.1274 g of AgNO_3_ ($C_{{\mathrm{AgNO}}_{3}}=0.75\;M$) solutions was added to the flask at the rate of 25 μL/s by injection pump. Then, 9 mL of EG solution containing 2.4201 g of AgNO_3_ ($C_{{\mathrm{AgNO}}_{3}}=1.583\;M$) solution was added into the flask immediately. Then the flask was sealed until the solution became glistening, indicating the formation of AgNWs. Upon the completion of the reaction, the reaction solution was transferred to an ice bath and allowed to cool. The products were collected by acetone sedimentation combined with centrifugation. The final products were dispersed in ethanol and stored at room temperature for further characterization. The influence of I^−^ was also explored in the mixture system of Cl^−^ and I^−^ using a similar procedure.

### 2.3. Density Function Theory Calculation

Density functional theory (DFT) calculations were performed by using the Vienna Abinitio Simulation Package (VASP 5.4.4, Hafner Group at the University of Vienna, Vienna, Austria). The kinetic energy cutoff of electron wave functions was set as 450 eV. The convergence of energy standard for electronic self-consistent processes was set to be 1 × 10^−6^ eV, and the convergence criteria for force in geometric structure optimization was set to be 0.015 eV Å^−1^. The adsorption models of different halogens on Ag(111) and Ag(100) crystal planes were constructed, respectively. After structural optimization, the adsorption energy of halogen in the structural model was calculated as follows:

$$E_{ads}=E_{slab}^{ads}-E^{ads}-E_{slab}$$

where $E_{ads}$ represents the adsorption energy of halogens on different crystal planes of silver and $E_{slab}$, $E^{ads}$, and $E_{slab}^{ads}$ represent the energy of the initial, halogen, and the adsorbed structure, respectively.

### 2.4. Characterization

The morphologies and microstructures of as-prepared samples were investigated by field emission scanning electron microscopy (FE-SEM, Verios G4, Thermo Fisher Scientific, Waltham, MA, USA) and transmission electron microscopy (TEM, Tecnai G2 F20, FEI, Hillsboro, OR, USA). The crystal structure of products was characterized by X-ray diffraction (XRD, D8 Advance, Bruker, Karlsruhe, Germany). The ultraviolet–visible (UV–vis) extinction spectra of the AgNWs suspensions in ethanol were recorded by an optical spectrometer (UV2600i, Shimadzu, Kyoto, Japan). The surface chemistries were characterized by X-ray photoelectron spectra (XPS) on an X-ray photoelectron spectrometer (ESCALAB 250 Xi, Thermo Fisher Scientific, Waltham, MA, USA) with results being calibrated by C1*s* at 284.8 eV. The zeta potential was measured by Zeta potential analyzer (Zetasizer-Nano-ZS90, Malvern, UK). The specific surface area was tested using the nitrogen adsorption and desorption isotherms on a specific surface area analysis instrument (ASAP 2460, Micromeritics, Norcross, GA, USA), with the surface area being calculated by the Brunauer–Emmett–Teller (BET) method. The diameter and length of 200 wires were calculated from the SEM images for each sample, with the aid of the Nano Measurer 1.2 software (Fudan University, Shanghai, China). In particular, in order to measure the actual length of the wire, the final products dispersed in ethanol were subtly diluted for preparing SEM samples, so that most of the boundaries between nanowires could be clearly distinguished. Moreover, some bended nanowires are divided into several segments and each segment was separately calculated to decrease the measurement errors.

## 3. Results and Discussion

### 3.1. Growth Characterization of AgNWs in Halogen-Containing Systems

**Figure 2:**
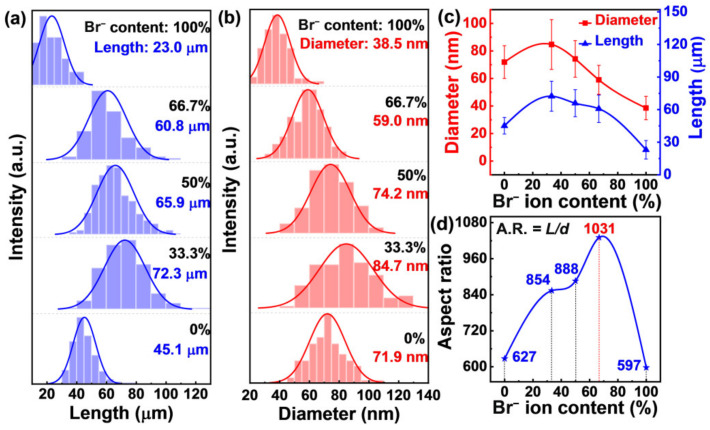
The histograms of length (**a**) and diameter (**b**) of AgNWs with Br^−^ ion content varied from 0% to 100% in total halogens. Changes in AgNWs diameter and length with Br^−^ ion content are summarized in (**c**). The aspect ratios of AgNWs with Br^−^ ion content are summarized in (**d**).

The co-addition of Br^−^ and Cl^−^ results in the higher aspect ratio of AgNWs than both single Br^−^ and single Cl^−^ systems. The total halogen ions content was kept the same for all experiments by controlling the added amount of FeCl_3_ and FeBr_3_. Typical XRD patterns and TEM images are shown in Figures S1 and S2, proving the successful formation of AgNWs. As shown in Figure 2a, the average length of AgNWs was only 45.1 μm for single Cl^−^ system (labeled as 0% in Figure 2), which is much lower than 72.3 μm of 33.3% Br^−^, 65.9 μm of 50% Br^−^, and 60.8 μm of 66.7% Br^−^. The results imply that the co-addition of Cl^−^ and Br^−^ can facilitate the growth of the Ag(111) crystal planes, resulting in AgNWs with larger length.

The diameter shows a similar trend as shown in Figure 2b,c. Specifically, the average diameter of AgNWs at 66.7% Br^−^ system is much lower than the single Cl^−^ system (59.0 nm vs. 71.9 nm), pointing to restrained growth of the Ag(100) crystal plane when 66.7% Cl^−^ was replaced by Br^−^. It should be mentioned that both the length and diameter present the smallest values for single Br^−^ system in all tested samples, showing that the growth of both the Ag(111) and Ag(100) are the slowest. Resultantly, the AgNWs with the highest value of 1031 was obtained in the 66.7% Br^−^ system.

**Figure 3:**
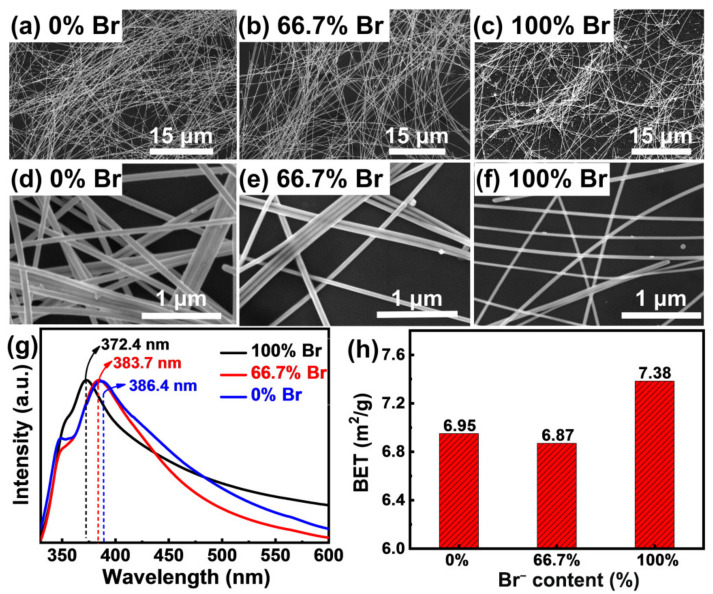
(**a**–**f**) SEM images of purified samples of AgNWs obtained with different Br content. (**g**) UV–visible extinction spectra of the final products. (**h**) The specific surface area of AgNWs.

The above results are further manifested by the morphology observations and UV–vis extinction spectra as well as the specific surface area comparisons. As shown in Figure 3a–c, the low-magnification SEM images present a much shorter length of AgNWs for 100% Br^−^ system (Figure 3c) than that of both 0% Br^−^ system (Figure 3a) and 66.7% Br^−^ system (Figure 3b), further proving that the growth of Ag(111) crystal plane is restrained in the single Br^−^ system [29]. Correspondingly, the inhibited growth of the Ag(100) crystal plane in both 66.7% Br^−^ system and 100% Br^−^ system were again validated by the high-magnification SEM images (Figure 3d–f) [29], as proved by the much thinner nanowires for these two cases when compared with that of 0% Br^−^ system (Figure 3d). The small difference of distribution density for AgNWs in different SEM images is mainly caused by the discrepancy of the dispersed concentration of AgNWs during the SEM sample preparation process, rather than the difference of product yield in various conditions. The yield of AgNWs is located in the range of 30~60%, depending on the added halogen ions, being consistent with the reported results [2,29,36]. The above characteristics for the diameter of the AgNWs were also revealed by the UV–vis extinction spectra (Figure 3g). In the UV–vis extinction spectra of AgNWs, lower wavelength of the absorption peak means thinner nanowire [2,43], which clearly indicate the diameter obeys the order of 0% Br^−^ > 66.7% Br^−^ > 100% Br^−^ (Figure 3g), again validating the inhibition effect of Br^−^ for the growth of the Ag(100) crystal plane. In addition to the UV–visible extinction spectra, the comparison of the specific surface areas of AgNWs also embodies the above morphology features. The specific surface area (*S*) of AgNWs can be determined by its length and diameter ($S=\frac{K}{L}+\frac{K}{d}$, where *K* is a constant, *L* is the length of AgNWs, and *d* is the diameter of AgNWs). When the average length and diameter were substituted for calculation, the specific surface area of AgNWs could be ranked as *S*(100% Br^−^) > *S*(0% Br^−^) > *S*(66.7% Br^−^), which is consistent with the detected BET values in Figure 3h, again manifesting the reliability of the above-mentioned morphology characteristics.

The influence of I^−^ on the growth of AgNWs is also explored in the mixture system of Cl^−^ and I^−^. In the I^−^ ions dominated conditions (50–100% I^−^), Ag nanoparticles are the only products without the generation of AgNWs. The generation of Ag nanorods (only ~0.74 μm) with an extremely small aspect ratio of 15.1 is observed only when the I^−^ is as low as 33.3%. The results indicate that the existence of I^−^ can restrain the formation of AgNWs, being consistent with the reported results [44,45].

In a word, the growth characteristics of AgNWs in different halogen-containing systems were understood. Compared with single Cl^−^ system, replacing 66.7% Cl^−^ with Br^−^ can promote the growth of Ag(111) crystal plane while the inhibition of the growth of the Ag(100) crystal plane at the same time, contributing to substantially increased aspect ratio of the AgNWs from 627 to 1031.

### 3.2. Analysis of Surface Physicochemical Characteristics of AgNWs

**Figure 4:**
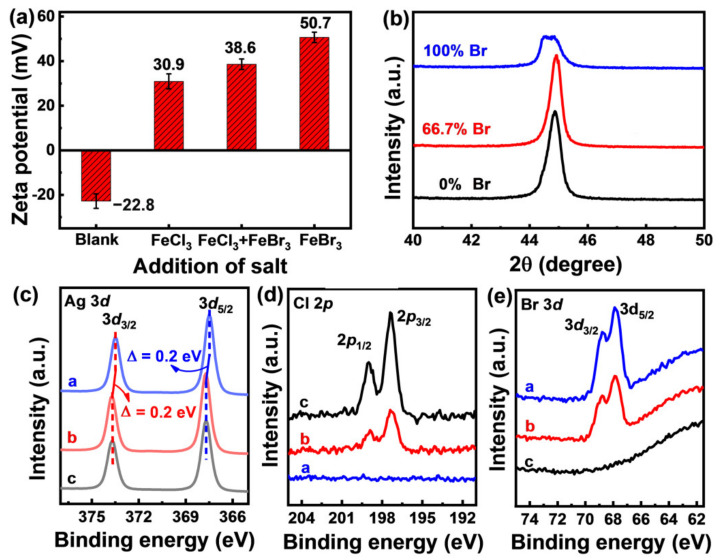
(**a**) Zeta potential of as-synthesized AgNWs in different solutions. Equal amount of halogen ions are added in FeCl_3_ solution, FeCl_3_–FeBr_3_ mixed solution, and FeBr_3_ solution; (**b**) XRD pattern of as-synthesized AgNWs. The high-resolution XPS regions of Ag 3*d* (**c**), Cl 2*p* (**d**), and Br 3*d* (**e**) for AgNWs synthesized at different halogen compositions: the blue line, red line, and black line represent the synthesis conditions of 100% Br^−^, 66.7% Br^−^, and 0% Br^−^, respectively.

Physicochemical analysis reveals that Cl^−^ and Br^−^ show huge differences of adsorption behavior on the surface of the AgNWs. As shown in Figure 4a, in FeBr_3_ solution, the zeta potential of added AgNWs is 50.7 mV, which is much higher than 38.6 mV in the FeCl_3_–FeBr_3_ mixed solution and 30.9 mV in FeCl_3_ solution. The decrease of zeta potential is due to the adsorption of halogen anions (Cl^−^ or Br^−^) on the surfaces of AgNWs, which means that the least amount of halogen is adsorbed in FeBr_3_ solution as revealed by the highest zeta potential value (Figure 4a). The adsorbed halogen anions can propel the accumulation of Ag^+^ on the surface of AgNWs because of the extreme low solubility of silver halides [2,35,36,43,46]. The low amount of Ag^+^ on the AgNWs surfaces in single Br^−^ system lead to slow growth rate, resulting in very thin and short nanowires (see Figure 3a–f) as well as weak crystallinity, as revealed by the lowest peak intensity in the XRD pattern of AgNWs obtained in the 100% Br^−^ system (Figure 4b). The similar peak intensity for AgNWs obtained in 0% Br^−^ system and 66.7% Br^−^ system (Figure 4b) are consistent with the low zeta potential gap between FeCl_3_ solution and FeCl_3_–FeBr_3_ mixture solution (Figure 4a), also manifesting the influence of halogen ions adsorption on the growth of AgNWs. The change of zeta potential of AgNWs from the negative value (−22.8 mV) in blank solution to positive values (30.9~50.7 mV) in iron halides-containing solutions is due to the adsorption of Fe^3+^ cations.

The surface chemistry analysis by XPS technique also reveals the different adsorption behavior between various preparation systems. As shown in Figure 4c, compared with the AgNWs in 100% Br^−^ system (blue line), positive shifts of 0.2 eV of Ag 3*d* peaks were observed for AgNWs in the 66.7% Br^−^ system and 0% Br^−^ system. The results indicate that the AgNWs in the later two cases are inclined to lose more electrons, meaning stronger interactions with the surface halogen ions (Figure 4d,e) [47,48].

In this system, the added Fe^3+^ can be reduced to Fe^2+^ by ethylene glycol, which can efficiently remove the adsorbed oxygen atoms, facilitating the formation of multiply twinned seeds. The generated multiple–twinned seeds are critical for forming AgNWs [41]. Similarly, other cations, such as Cu^2+^ and Mn^2+^, can also induce a similar effect, also being used for promoting the formation of AgNWs [35,49,50]. The cations of Na^+^ and K^+^ cannot induce such an effect, therefore needing a deoxygenation procedure by inert gas during the production of AgNWs [29,49,51].

The physicochemical characteristics on the surface of AgNWs play an important role in determining the growth behavior of AgNWs. Replacing Br^−^ with Cl^−^ can promote the adhesion of more halogen ions to the surfaces of AgNWs, contributing to faster growth and higher crystallization degree. Additionally, the interaction of AgNWs with the surface halogen can be enhanced by tuning the relative ratios of added Br^−^ and Cl^−^.

### 3.3. DFT Calculation for Understanding AgNWs-Halogen Interactions

**Figure 5:**
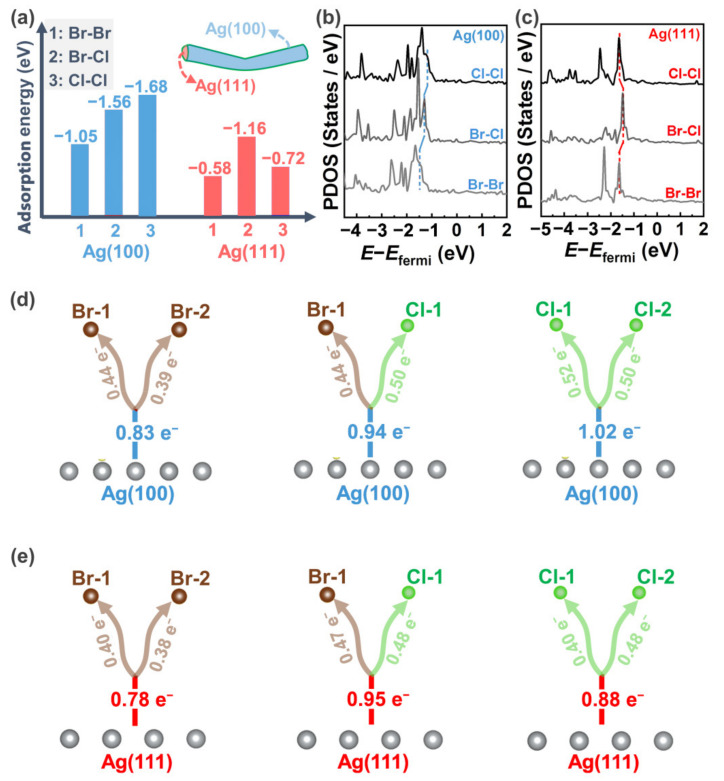
(**a**) Comparison of adsorption energies of halogens on Ag(100) and Ag(111) crystal planes. (**b**,**c**) Partial density of states (PDOS) of silver atoms on Ag(100) and Ag(111) crystal plane. (**d**,**e**) Bader charge distribution diagram of Ag(100) and Ag(111) crystal planes. The insets in (**a**) illustrates a schematic diagram of a single AgNWs.

The interactions between AgNWs and halogen ions were further revealed by DFT calculations for further understanding the preferential growth of specific crystal planes in different halogen-containing systems. As shown in Figure 5a, the adsorption energy of halogen ions on Ag(111) crystal plane is the strongest (−1.16 eV) for the Br–Cl mixture system (labeled as Br–Cl), which implies the largest number of Ag^+^ is accumulated [2,35,36,43,46]. Correspondingly, the Ag(111) crystal plane of AgNWs in Br–Cl system possesses the fastest growth rate among the three halogen-containing systems, being consistent with the morphology analysis in Figure 3a–c and length trend in Figure 2c. The adsorption energy of halogen ions on the Ag(100) crystal planes (Figure 5a) obey the order of single Cl^−^ system (labeled as Cl–Cl, −1.68 eV) > mixed Br–Cl system (labeled as Br–Cl, −1.56 eV) > single Br^−^ system (labeled as Br–Br, −1.05 eV), which means the diameter size of the AgNWs follows the similar trend, being in accordance with the diameter characteristics in morphology results (Figure 3d–f) and UV–vis extinction spectra (Figure 3g).

The above results are further revealed by the partial density of states (PDOS) and Bader charge distributions. As shown in Figure 5b, the density state of silver atoms on Ag(100) crystal plane in single Cl^−^ system (labeled as Cl–Cl) is the closest to the fermi energy among the three calculated models, which means that the silver atoms on Cl–Cl model is the most active for adsorbing the halogen ions. Similarly, the density state of silver atoms on the Ag(111) crystal plane in the Br–Cl mixture system (labeled as Br–Cl) is the closest to the fermi energy among the calculated three models (Figure 5c), which indicates that AgNWs in the Br–Cl mixed solution possesses the highest growth rate in the length direction, being in accordance with the results in Figure 2c. The Bader charge distributions also reveal the above results (Tables S1 and S2, Figure 5d,e). Among the three calculated models, the electron number being transferred from the Ag(100) crystal planes to the halogen atoms is the largest for the single Cl^−^ system (1.02 e^−^, Figure 5d), which means the single Cl^−^ system promises the strongest adsorption of Ag(100) to the halogens, resulting in the fastest growth of diameter. Correspondingly, the electron number being transferred from the Ag(111) crystal planes to the halogen atoms is the largest for the Br–Cl mixed system (0.95 e^−^, Figure 5e), which indicates the Br–Cl mixed system results in the strongest interactions between the Ag(111) and halogen atoms, contributing to the highest growth rate of the length.

**Figure 6:**
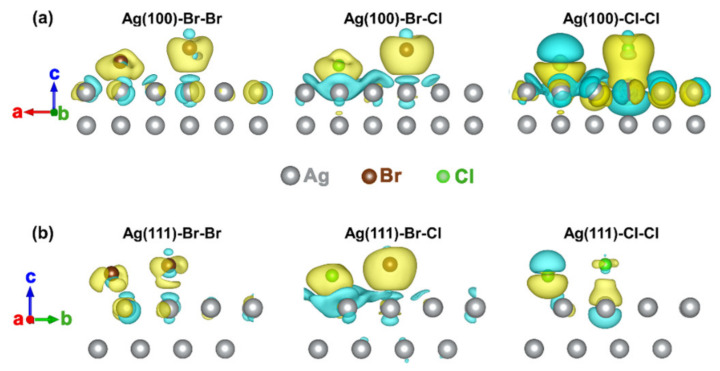
(**a**,**b**) Isosurfaces of local charge density difference for different models (yellow and cyan denotes 5.2 × 10^−3^ and −5.2 × 10^−3^ e^−^ per Å^3^ isosurfaces, respectively).

The accumulation of electrons around the halogen atoms are also revealed by the local charge density difference. As shown in Figure 6a for the Ag(100) crystal planes, more electrons are accumulated around the halogens in the Cl–Cl model (yellow isosurfaces) than that in both the Br–Br model and Br–Cl model, which implies the single Cl^−^ system results in the strongest interactions between halogen atoms and the Ag(100) crystal plane, being consistent with the discussions in Figure 5. Correspondingly, the mixed Br–Cl system contributes to the most amount of electron accumulations around the halogen atoms on the Ag(111) crystal plane surface, as shown in Figure 6b, being in accordance with the adsorption energy and Bader charge distributions in Figure 5. The above results again embody that the single Cl^−^ system promotes the fastest growth of the Ag(100) crystal plane, while the Br–Cl mixed system contributes to the highest growth rate of the Ag(111) crystal plane.

Conclusively, compared with the single Cl^−^ system, the co-addition of Br^−^ and Cl^−^ can enhance the adsorption of halogens on the Ag(111) crystal planes while weaken the adsorption of halogens on the Ag(100) crystal planes. The adsorbed halogen ions contribute to the concomitant accumulation of Ag^+^ due to the extreme low solubility of silver halides. Resultantly, the aspect ratio of AgNWs prepared in the Br–Cl mixed solution is much higher than that in both single Br^−^ solution and single Cl^−^ solution. Correspondingly, the adsorption energy of halogen atoms on both the Ag(100) and Ag(111) planes are the lowest in the single Br system, resulting in AgNWs with the thinnest diameter and shortest length. The above analysis was further embodied by the PDOS and Bader charge distributions as well as the isosurfaces of the local charge density difference.

## 4. Conclusions

The size regulation and mechanism analysis of AgNWs mediated by halogen ion composition were systematically conducted. The co-addition of Br^−^ and Cl^−^ can greatly increase the aspect ratio of the AgNWs. When Br^−^ accounts for 66.7% in total halogens, the aspect ratio of AgNWs reaches to the highest value of 1031. The physicochemical analysis, including zeta potential testing and XRD analysis as well as XPS results, all indicate that the morphology and size evolution of AgNWs growth are highly dependent on the interactions of halogen ions with the AgNWs. The DFT calculation results demonstrate that the co-addition of Br^−^ and Cl^−^ contributes to the highest adsorption energy of −1.16 eV for halogen atoms on the Ag(111) crystal planes, facilitating the strongest interactions between halogens and Ag(111) crystal planes. On the contrary, compared with the single Cl^−^ solution, the co-addition of Br^−^ and Cl^−^ decreases the adsorption energy of halogen atoms on the Ag(100) crystal planes from −1.68 eV to −1.56 eV, with the interactions between halogen atoms with the Ag(100) crystal planes being largely weakened at the same time. Resultantly, the enhanced preferential growth of Ag(111) crystal planes as well as the restrained growth of the Ag(100) crystal planes contribute to a much increased aspect ratio. The results herein can provide new insights for understanding the morphology and size evolution during the AgNWs preparation in the ethylene glycol system.

## Data Availability

The data presented in this study are available on request from the corresponding author.

## Supplementary Materials

The following supporting information can be downloaded at: https://www.mdpi.com/article/10.3390/nano12152681/s1, Figure S1: XRD pattern of AgNWs synthesized with different halogen composition. All the peaks could be indexed to fcc silver. The blue line, red line, and black line represent the synthesis conditions of 100% Br^−^, 66.7% Br^−^, and 0% Br^−^, respectively; Figure S2: TEM images of AgNWs obtained with 66.7% Br^−^ and 33.3% Cl^−^. (a,b) Low magnification image of AgNWs; (c) high magnification image of AgNWs; (d) a zoom-in TEM image taken from the marked square area in image (c), demonstrating the lattice fringe with spacing of 0.237 nm; Table S1: The number of valence electrons for different halogens on Ag(100) crystal face in AgNWs; Table S2: The number of valence electrons for different halogens on Ag(111) crystal face in AgNWs.
