# Supplementary material for: Mechanism Understanding for Size Regulation of Silver Nanowires Mediated by Halogen Ions
Source: Nanomaterials (Basel). 2022 Aug 4;12(15):2681. doi: 10.3390/nano12152681 (PMC9370693; doi:10.3390/nano12152681)
Supplement: Supplementary file 1 [file nanomaterials-12-02681-s001.zip › nanomaterials-1830929-supplementary.pdf]

nanomaterials

Supplementary Materials

Mechanism Understanding for Size Regulation of Silver Nanowires Mediated by Halogen Ions

Ni Xiao [1], Yinan Chen [2], Wei Weng [2,3], Xiaopeng Chi [2,3], Hang Chen [4,5], Ding Tang [4,5] and Shuiping Zhong [2,3,4,5,*]

1 School of Materials Science and Engineering, Fuzhou University, Fuzhou 350108, China; 18396310297@163.com
2 Zijin School of Geology and Mining, Fuzhou University, Fuzhou 350108, China; chenyn0926@163.com (Y.C.); wengwei198912@163.com (W.W.); xiaopengchi@fzu.edu.cn (X.C.)
3 Fujian Key Laboratory of Green Extraction and High-Value Utilization of Energy Metals, Fuzhou University, Fuzhou 350108, China
4 Zijin Mining Group Co. Ltd., Shanghang, Fujian 364200, China; 18859053970@163.com (H.C.); tang_ding@163.com (D.T.)
5 State Key Laboratory of Comprehensive Utilization of Low Grade Refractory Gold Ores, Shanghang, Fujian 364200, China
* Correspondence: zspcsu@163.com; Tel.: +86-15280385768

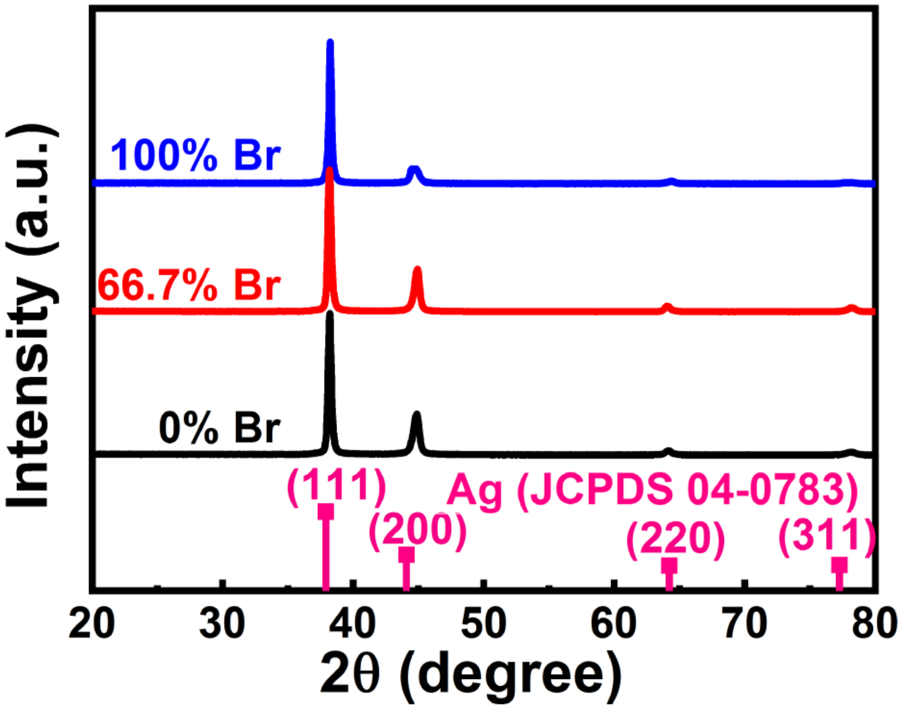

Figure S1. XRD pattern of AgNWs synthesized with different halogen composition. All the peaks could be indexed to fcc silver. The blue line, red line and black line represents the synthesis conditions of 100% Br^-, 66.7% Br^- and 0% Br^-, respectively.

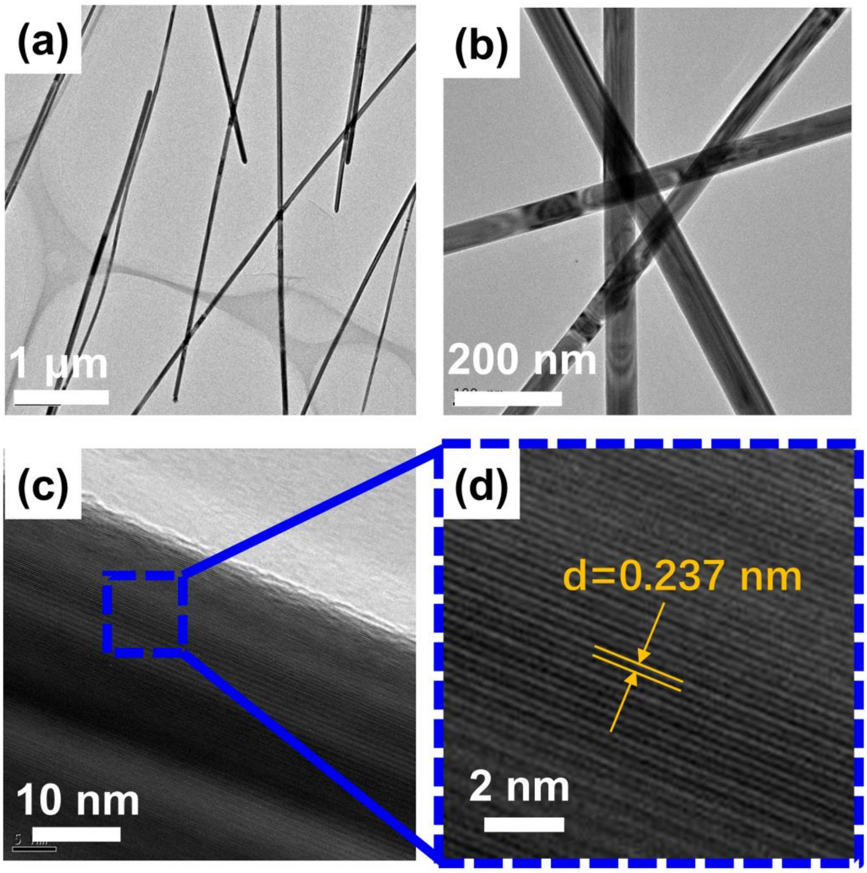

Figure S2. TEM images of AgNWs obtained with 66.7% Br^- and 33.3% Cl^-. (**a**,**b**) Low magnification image of AgNWs; (**c**) high magnification image of AgNWs; (**d**) a zoom-in TEM image taken from the marked square area in image (**c**), demonstrating the lattice fringe with spacing of 0.237 nm.

Table S1. The number of valence electrons for different halogens on Ag(100) crystal face in AgNWs.

Model	Atom	Actual Electron Number	Number of Valence Electrons	Number of Electrons Gained	Total Number of Electrons Gained
Br–Br	Br1	7.4419	7.0000	0.4419	0.8347
	Br2	7.3928	7.0000	0.3928	
Br–Cl	Br1	7.4384	7.0000	0.4384	0.9490
	Cl1	7.5106	7.0000	0.5106	
Cl–Cl	Cl1	7.5147	7.0000	0.5147	1.0218
	Cl2	7.5071	7.0000	0.5071	

Table S2. The number of valence electrons for different halogens on Ag(111) crystal face in AgNWs.

Model	Atom	Actual Electron Number	Number of Valence Electrons	Number of Electrons Gained	Total Number of Electrons Gained
Br–Br	Br1	7.3987	7.0000	0.3987	0.7828
	Br2	7.3841	7.0000	0.3841	
Br–Cl	Br1	7.4702	7.0000	0.4702	0.9500
	Cl1	7.4798	7.0000	0.4798	
Cl–Cl	Cl1	7.3952	7.0000	0.3952	0.8801
	Cl2	7.4849	7.0000	0.4849	
